# Supplementary material for: Full-Length Transcriptome Analysis of Alternative Splicing and Polyadenylation in the Molecular Regulation of Labor Division in Apis cerana cerana
Source: Int J Mol Sci. 2025 Aug 14;26(16):7859. doi: 10.3390/ijms26167859 (PMC12387084; doi:10.3390/ijms26167859)
Supplement: Supplementary file 1 [file ijms-26-07859-s001.zip › File S1.pdf]

File S1. Full sequence of XM_017060180.2, LOC107998857_novel04, LOC107998857, LOC108004007 LOC107998473, LOC107998988 in mRNA.

>XM_017060180.2

ATGTTTCCTGTGTGTACGTTAGCTGCCAGTCGACAAATCTTCTTTTTGCACGAGTGTGTGCACGTCGAA
CGTAAACGCGTTGTCGACAACCTGGAGTATAACCTCAATATTTATAGTAAAATGTTCAAAATCAAAGAA
ATCTCTTAAAAACATTTTCGTTCTTTCTTTTTCTAATTGTTTTCTAGCAATTTAGATTATGTTATATTCAAT
TGGAGTACATGCAGTAACTCGCATATTATTCTCATGAATTTCAAGTATTTGTCGTTTTTAAACATTATCAA
AAAAGATAATTGCATCCACAAATTGAGATTTTAAGTTTCAAGTATCTCTCATTTTTGGCCCGATTGCCGT
TTCATCGTTTAAATTGAATTTTTCGATAAATGTTTTTTATCAGCATGAATCGAATATATTATTTATTAGAGA
TTAAAGAAATGTCATCGACATGGGTGCTGTTGTGGATGTCGTTACGGCCCTGGAAAAGACGGCGATCA
CTAAAGAAGTGCTTGAGGTTACAAGACTTGGAAAATATATTAATGAACTTCGACGTAAAACAAGTAAT
GATGCTTTGGCAAAACGAGCAAAGGATTTGGTCCGGCGATGGCGTGATATGGTCTTACCTTCTGCTCAT
TCTACTCCACAACCTACACCAGCAGATACAGCACCACCTGCTCTTAATGGAGCAAAACCACATAGTCC
TGCATTAAGATGTTTTAAACCACAAAGTCCAGCATTAAGAGGTCTGATACCACCTCAGAGTCCATTATT
AAGGGATAGTACTCCACTTAGAGTACTTAGTCCAGTTCTATCTGTGCATAGTGATCATTCACATTCTCCT
AATGCATCATCAAATAAACAGTCAATTACAGTAACCAGCAATCACAGAACAAGTTCTGATGTGCCACC
TATGCTTGGTTCTTCAAGCCAACATCATTCGACAGAAGCAGTGCCACGAACTCACAGTTCAAACAAAC
GTCTCCGAAAAGATGATTTTAAGGACCAACACAATTATCAACACCATGATTCAGACTCAATCACCGAA
AGTGAACCCTTTGTTAAAAAACAACGTCTAAATGGCGAAAATATAAGTGGTAATTTAAATATGCAAGTG
CCTAGCCCCATATTTAAAGAAAGAATCTCTGATCAGTTTACGGAGTCTTCCACAGATCCTGATAATTCA
GGGCCAAAGAAACGTGGTAGAAAAAAAGGCAGTAAATCTGTAAAAAAACAACCAATTTTAGAAGATC
GTGTAAAAGAAAAACTAGCTAGTATTTCAAGGAATCCTAAATTAAAAACAACTCAAGAATTGCTGGCT
GATCTACGGGCACGCGGCACCAATTCTAGCATTAATTCAAGTGCTCTACCTAGTCAAAGTGTAGAATCA
TCCAGCATGGAAGAAGTTCTGAGAAATAGCAACGAACAAGTATCAAAGTTCCTTCGTTGTCCTCAGAA
TAATTTATCTCATAGAAATACAGTTTCTGAAGTCTCAGCAGAAAGTCGGTTAAAACCAACAGAAAACT
GTCATGATGTACCATCTAAGTGTGACGAATCAGGTGTAGTGTTCAAAGAAAAACCAGTTACAAATGTT
CAAAAAAATCAACCAGAACAGTGTCGAGATCTCACAGTTGAAGAAATATTGGCTAAGTTACCACCTAT
AGATCCAAATTCAATAGATTGGAGTGAGTGTGAAATTGAATCAACTGAAGATCAAAATAGTTCTGAATA
TCCACCTAGACAAGTTACTGCAGAGGACATAGAAAGGTTACATACACAGTGTGTAGAAGGACTGAATG
GAAACTTCCAACCAAAACTATCATCTTTAACTTCGTCTTTTAATGAGAATCAGAGAAAAGAAGATGTG
ACAGATGTCAATGGTGTAAACAATGTGTATAGCCAACCTGATGAAGAGTTTCGAGAATGGCATCAGAT
GCTAGCAAGGCCCAGTTACAATGGCCAGATCCTCCACATATTGCCTTATGTTATTATTGATTAGTAATTTT
ATTATCTTACTATTATCCTGAAACTTGTGAAGAATCCCTGCAAATATTAAAGAGATTGATCAATGATCAG
TCTATTAAATCGATTTCTATTGC

>LOC107998857_NOVEL04

AGTAGTCTTCTTAACAAGGGTTACACTATAATGAATACCCTAATCGTATTATTGGGAATCCTAACTACGG
TGATTGCCGTTCCGGCTCCTTTTCCACATGGAAAACTTGTAACTTACAAATACATCGCCGATGTGAATG
CTGGTGTCGAGTCATCTTTATTCGTATCCAATTATGGCATCGAATGTTTACTTAACGTACAACATGTCAC
CGATACAAATTCGAAGAATACTTATTATGTTAATTTGACCAATGTGAAATATGGACTACATAATGGATTT
GTGAAACATTACGAATCATTGTCTGTTATGGAATCGATCCCCGACGTGGCAAATGCGATTCTGAATCCT
TTCCTCGTCGTTTATGACGAAAATGGACACCTTAAAGGTGTAAAATTTATGGAAAACGAGCCAGGATG
GTCAAGAAACATGAAAAAGGGTATCGTTTCTATGCTTCAATTGGACATGACTAATATTCGAGTTCAAAC
TCCGATAAAATCACATAGTTTCATAACTCACGAGGAAACCGTCCACGGTACTTGCCAAGTAGCTTATAA
TGTTCATCCATTGGACCATGTAGGATCTAACAAAGAATTCGTCGTTACAAAGATTCACGAATCGAAGAA

TTGTACTCGTTTTTCTTACCACGAGATGAGCCATCTTGATTCTGAGAAATGTCACGTAGATGAATGGGA
TGACGTGACCACTGCTAGTAGACGCATTTTCGTGGTAGAAAATCAAGACAATGAAGTTCTTATAAGAA
AATTAATTAGTCATGGAATGATCAACTATCTACCGTGGGCGGCCGAATCAGAGGCGCATCACCTTCTGA
CCAACCAAACGTTGCATCTCAAAGACGTTGTCTCTGTATCCGAGTCACACTTATCAGACATCGTACGAT
TAAATCCTTCAATTATCGAAAATGTTATCTACGATGAACCTACGACTTCGTACGTTCCTCAAGCTGACGT
AGACGTAACCCATGGTCGACATATCGTCAAGCTCGATGATCTTATTATAAAATTGAGAAAGATGTTGGA
CGAGGTTGCTGGTTATCTCAAAGAAAGCCAAACCGACAAGAAACAATCGGATTGGAAACACGATCAA
ACGATCAATAGGATTTTACAAGTTATGAGTTACATGGACGTCGCATCATTGGAGCAAGTGTACAGCGAG
ATTCAAAATGGTAAAGATGCCAATGAGATAGCGAAGAGAAATATTTTCCTCGGAATACTGTCAAGTGTA
GGAACCCCAGCTGCCTGCTTTTTCACGCGAAACGTAGTTCGGACAAAGAGCGTTCCAAAATGGAATG
CAATTATGATGCTTGGAAATCTTGCGATGCATGTGAAGGTTCCTTCGAAAAAACTGCTTGTGCAGATGG
AGGAACTTCTTAACTTAGACGATTCTGTTCCCGTGGAAGTTAAGGAAGCGAGTATATTCTGTTTTGCTA
CATTGATTCGTAAAACATTTGAGCACGAGGAGACTGGAGCTATAGATCCTCTTTTGGATAAATACTTGC
ATCACTTCATGGAACATATTAGAAACGAACCGACGCATCATATGAAGATGGTATACATGATGGCAATGA
AAAACGTAAGATTAACTCAGATTCTGAAGTTTTTGGAGCCTATCATCCGCGGCGACGAAGTTGTATCCG
ATAAACCGCACAACATTCGCGCACAAGCTATTTGGATCATGAAAAACGTAGTCTTCGAACATCCCCGTT
ACTGTTACAACTTACTCTGGCCGGTTCTAACCGATGTTACCCTTCCAACAGCAGTTAGAATAGCCGCGT
TTGACGTCTTGATGAACGAAACGCCTCAATTAGGACGGTTTATAAATGTTTATTGGTTGATGGTTTACG
AAAAGAACGAACATCTCTATAATTATTACGTGGATACCATCAAAGGACTTGCTACCTCGGTAGATCCTT
GCCTGATGCAGGCTCGAGAAATGGCTAAGAAATTATTAAAGATCGTTCGAGTAAGGAACGTTACCGGT
CCATTATCGCGCAAATTTTATGTCGATTACGTGGATAATAAGTATGAATATAATGAAAGAGTGAAAGGTT
CCTTAATTGTGGATCACACATCTGGCTTGCCGTACGTTGGTTCGATCGAACATATCGCATCTGTTAAAGC
ACGCAAACCTGTTACTAAATTAGGAATCCACTGGAATATAGATGGTTTGAGCGAGATTGTTAAGGATAT
CGATGAAAATGTGTTCGGCAATGTCGTACAAGCGATTAAAAACGACAATGTGAAAAATGTATTAATTTC
AGCAGCTAAGGTTATGCCGAAAATTAAAGATGTTAACGTTAATGTCATTTTAACAATGAACGACAATGT
TGTTTCGATATTCCATTGCACCAAGAATAATTGGCAGAAAATTCTCAATGAATTGAAACAGTGGAAAAA
ATTGATCACCGAATACATGGGTGTTATTAACTGGCAGACTGTTTTATATCACGATCATTACGAAATGCAC
GTACCCACTGATCTTGGTGTACCAGCCATTTTGGCAACGAAAATACCCTCTTTATTCTCCCTCGTAGGA
AGCTTTGAAGTCTCTGAAGATAAGAATCTTTTAATCTTGAAACCAAAAATTAAATACCAGCAATGGATG
CACGGCGAACACACGATGTCAATTTACAATCCTGTCGTTGATGTCTGGCACTCTGTTCATAAGACATCG
TCTTTCGATATTGTTACACCATGTGAAATGAGCATTGGCTGGAACTGGAAAACGAAGAGTCTTAAAGT
TACATGGCCAAGATTGCCAGTTTCGGATTTCTCGGTTGCGGGAATATCGATTCACGGTAAAAATTATATC
ACCGTTTTAAACGATGAGCATGATCTGCTGAAAAAGTTTTGTTCTACTTGTCATCATTATGAAGTTGTTA
CCAATAACGTCAATTTGAAAACGTACGAGAATACTTTCGATTCAAAGGATATTGGAACTCGATTTAAGA
TGCAATATCATTGCGACAGTGACTTTATTCCGGTTTCTTTTGTGGATGAATGGCTTATAGGACTTTCAGA
GTCAAAGACAATCGAAAACAGTATGATGCAGGCTGTTATAGCGATCCGTCATAAAATAATGAGCGATAT
AATTTTGGGACAAGGAAAAAGTTGTTCTCTCGTGTGTAAAGCTGAACCAAGTATCGTTTATCCTACTTC
AGTGATCGACTTAATTGGAAAAGTTAGCATTCAAGATTTTCACGAGACACAAAAAATGGATCTTTTAC
GTAGTAAGAGAATCGATATTCGTGGAAATTTAAATGCTAAAGCAGCTTCCACTAATGAATCTATAAGAC
AATGGGATATGAATCTTAACATTGTATTGTCTCAGGGACATGTTAATAATAGTATGAGAATTATGATTACT
CGTACCATACCAGAAGAGAAAAATTTAAAGATATGTATTGAGGCACAGAATGAATATCCAGAGATTACT
GATGATTTATTAACAGTTAATGGAAAGAAGGAAACTAATACAAAAATGACGATTACTATGGGACAAAC
GAAAGAAGATAAGTGTGTACGCGATGAAATGGATGTCACAATCATCATGAAAGGCGAAATGTTAGAAG
AACAAAAGAATCAAATGAGCTATGACAGCATGTATATTGAATGTTACAATCAAAGTCAGAATCCTTTGT

TCCATACCAAGAGTTCGAAAGTTCCTAAGACTAAAGAATGCATTGAAGAAGATATACTTCATTCAACTT
TGAGGAAATATACTTTAGATATGACCGTAAGAAAGGTACCTCAGCAAATAATGTCGGCTGTAAATATAG
TTCATGATAAATTGCGTGCTGTTTTCTTCGATCATATGAAACATATTTGGAATCATGTACAGCCTGGAAA
CGCGAAGATTGTCTTAGAATTCCCATACTTGACATCCGTTCTTAATGTAGCTGTTACAACTCCGACACA
CAGTTACGAATTAGTTCAATTACCATTTGGTAATCCAGTTTGGAATATATGGATGAATAATATTCATTATT
CTACGTCAATATTGAATGATTATTTTAATGAAAAAATTAGATTGTGCACAGTTTATCCTCGACTTCTTAAT
CCTACTATTAATGATGCCATTACTATTACTTGGAATGGTATCATTCAATTTATCAGATCAGACGAATGGAT
CTTGATGTCTGGTGATCACATACATCATACGTATAGTATCTTTGTGAAATTGGTACAAAATAAGAAATTG
GCTTTAAGAGTTTATATTGGTGAACATGAAATGGAAATTATGCCTCTTGAAACAAAAGTATCTGTGAAA
ATAGATAATAAAATTATTGATGATTATCAAAAAGGTATTACAGTATCCGAGAACGAATCACGTTCTTATT
CTATGAGATTAACCACTGCTTATAATTATGTAGTAATTGATTCACAAACGATACCAGTTCATATTTTCTAT
AAAGTAGACAGTGTGACGGTTTCTCTGCATACTAATCTTCAGAGTCAAGTAACTGGAATCTGTGGATA
ATATGGAATGATGATGTAGTTACAAAGAATAAAAAAATAAAATTTTACTTTGGAAAATTTTTAATTTTTT
ACAAAGATTATCATTAACTTTTGTTTTTATTATAATATACGATATATTTGTATCATATTAATTATCAAAGAAA
AAGAAATGATGTGTT
>LOC107998857
TTTACAGATTTTTCGCCATATAAAAGGAGAATGCAGCACAGCAGCGTTTAGTAGTCTTCTTAACAAGGG
TTACACTATAATGAATACCCTAATCGTATTATTGGGAATCCTAACTACGGTGATTGCCGTTCCGGCTCCTT
TTCCACATGGAAAACTTGTAACTTACAAATACATCGCCGATGTGAATGCTGGTGTCGAGTCATCTTTATT
CGTATCCAATTATGGCATCGAATGTTTACTTAACGTACAACATGTCACCGATACAAATTCGAAGAATACT
TATTATGTTAATTTGACCAATGTGAAATATGGACTACATAATGGATTTGTGAAACATTACGAATCATTGTC
TGTTATGGAATCGATCCCCGACGTGGCAAATGCGATTCTGAATCCTTTCCTCGTCGTTTATGACGAAAA
TGGACACCTTAAAGGTGTAAAATTTATGGAAAACGAGCCAGGATGGTCAAGAAACATGAAAAAGGGT
ATCGTTTCTATGCTTCAATTGGACATGACTAATATTCGAGTTCAAACTCCGATAAAATCACATAGTTTCA
TAACTCACGAGGAAACCGTCCACGGTACTTGCCAAGTAGCTTATAATGTTCATCCATTGGACCATGTAG
GATCTAACAAAGAATTCGTCGTTACAAAGATTCACGAATCGAAGAATTGTACTCGTTTTTCTTACCACG
AGATGAGCCATCTTGATTCTGAGAAATGTCACGTAGATGAATGGGATGACGTGACCACTGCTAGTAGA
CGCATTTTCGTGGTAGAAAATCAAGACAATGAAGTTCTTATAAGAAAATTAATTAGTCATGGAATGATC
AACTATCTACCGTGGGCGGCCGAATCAGAGGCGCATCACCTTCTGACCAACCAAACGTTGCATCTCAA
AGACGTTGTCTCTGTATCCGAGTCACACTTATCAGACATCGTACGATTAAATCCTTCAATTATCGAAAAT
GTTATCTACGATGAACCTACGACTTCGTACGTTCCTCAAGCTGACGTAGACGTAACCCATGGTCGACAT
ATCGTCAAGCTCGATGATCTTATTATAAAATTGAGAAAGATGTTGGACGAGGTTGCTGGTTATCTCAAA
GAAAGCCAAACCGACAAGAAACAATCGGATTGGAAACACGATCAAACGATCAATAGGATTTTACAAG
TTATGAGTTACATGGACGTCGCATCATTGGAGCAAGTGTACAGCGAGATTCAAAATGGTAAAGATGCC
AATGAGATAGCGAAGAGAAATATTTTCCTCGGAATACTGTCAAGTGTAGGAACCCCAGCTGCCTGCTT
TTTCACGCGAAACGTAGTTCGGACAAAGAGCGTTCCAAAATGGAATGCAATTATGATGCTTGGAAATC
TTGCGATGCATGTGAAGGTTCCTTCGAAAAAACTGCTTGTGCAGATGGAGGAACTTCTTAACTTAGAC
GATTCTGTTCCCGTGGAAGTTAAGGAAGCGAGTATATTCTGTTTTGCTACATTGATTCGTAAAACATTTG
AGCACGAGGAGACTGGAGCTATAGATCCTCTTTTGGATAAATACTTGCATCACTTCATGGAACATATTA
GAAACGAACCGACGCATCATATGAAGATGGTATACATGATGGCAATGAAAAACGTAAGATTAACTCAG
ATTCTGAAGTTTTTGGAGCCTATCATCCGCGGCGACGAAGTTGTATCCGATAAACCGCACAACATTCGC
GCACAAGCTATTTGGATCATGAAAAACGTAGTCTTCGAACATCCCCGTTACTGTTACAACTTACTCTGG
CCGGTTCTAACCGATGTTACCCTTCCAACAGCAGTTAGAATAGCCGCGTTTGACGTCTTGATGAACGA
AACGCCTCAATTAGGACGGTTTATAAATGTTTATTGGTTGATGGTTTACGAAAAGAACGAACATCTCTA

TAATTATTACGTGGATACCATCAAAGGACTTGCTACCTCGGTAGATCCTTGCCTGATGCAGGCTCGAGA
AATGGCTAAGAAATTATTAAAGATCGTTCGAGTAAGGAACGTTACCGGTCCATTATCGCGCAAATTTTA
TGTCGATTACGTGGATAATAAGTATGAATATAATGAAAGAGTGAAAGGTTCCTTAATTGTGGATCACAC
ATCTGGCTTGCCGTACGTTGGTTCGATCGAACATATCGCATCTGTTAAAGCACGCAAACCTGTTACTAA
ATTAGGAATCCACTGGAATATAGATGGTTTGAGCGAGATTGTTAAGGATATCGATGAAAATGTGTTCGG
CAATGTCGTACAAGCGATTAAAAACGACAATGTGAAAAATGTATTAATTTCAGCAGCTAAGGTTATGCC
GAAAATTAAAGATGTTAACGTTAATGTCATTTTAACAATGAACGACAATGTTGTTTCGATATTCCATTGC
ACCAAGAATAATTGGCAGAAAATTCTCAATGAATTGAAACAGTGGAAAAAATTGATCACCGAATACAT
GGGTGTTATTAACTGGCAGACTGTTTTATATCACGATCATTACGAAATGCACGTACCCACTGATCTTGGT
GTACCAGCCATTTTGGCAACGAAAATACCCTCTTTATTCTCCCTCGTAGGAAGCTTTGAAGTCTCTGAA
GATAAGAATCTTTTAATCTTGAAACCAAAAATTAAATACCAGCAATGGATGCACGGCGAACACACGAT
GTCAATTTACAATCCTGTCGTTGATGTCTGGCACTCTGTTCATAAGACATCGTCTTTCGATATTGTTACA
CCATGTGAAATGAGCATTGGCTGGAACTGGAAAACGAAGAGTCTTAAAGTTACATGGCCAAGATTGCC
AGTTTCGGATTTCTCGGTTGCGGGAATATCGATTCACGGTAAAAATTATATCACCGTTTTAAACGATGAG
CATGATCTGCTGAAAAAGTTTTGTTCTACTTGTCATCATTATGAAGTTGTTACCAATAACGTCAATTTGA
AAACGTACGAGAATACTTTCGATTCAAAGGATATTGGAACTCGATTTAAGATGCAATATCATTGCGACA
GTGACTTTATTCCGGTTTCTTTTGTGGATGAATGGCTTATAGGACTTTCAGAGTCAAAGACAATCGAAA
ACAGTATGATGCAGGCTGTTATAGCGATCCGTCATAAAATAATGAGCGATATAATTTTGGGACAAGGAA
AAAGTTGTTCTCTCGTGTGTAAAGCTGAACCAAGTATCGTTTATCCTACTTCAGTGATCGACTTAATTG
GAAAAGTTAGCATTCAAGATTTTCACGAGACACAAAAAATGGATCTTTTACGTAGTAAGAGAATCGAT
ATTCGTGGAAATTTAAATGCTAAAGCAGCTTCCACTAATGAATCTATAAGACAATGGGATATGAATCTTA
ACATTGTATTGTCTCAGGGACATGTTAATAATAGTATGAGAATTATGATTACTCGTACCATACCAGAAGA
GAAAAATTTAAAGATATGTATTGAGGCACAGAATGAATATCCAGAGATTACTGATGATTTATTAACAGTT
AATGGAAAGAAGGAAACTAATACAAAAATGACGATTACTATGGGACAAACGAAAGAAGATAAGTGTG
TACGCGATGAAATGGATGTCACAATCATCATGAAAGGCGAAATGTTAGAAGAACAAAAGAATCAAATG
AGCTATGACAGCATGTATATTGAATGTTACAATCAAAGTCAGAATCCTTTGTTCCATACCAAGAGTTCG
AAAGTTCCTAAGACTAAAGAATGCATTGAAGAAGATATACTTCATTCAACTTTGAGGAAATATACTTTA
GATATGACCGTAAGAAAGGTACCTCAGCAAATAATGTCGGCTGTAAATATAGTTCATGATAAATTGCGT
GCTGTTTTCTTCGATCATATGAAACATATTTGGAATCATGTACAGCCTGGAAACGCGAAGATTGTCTTAG
AATTCCCATACTTGACATCCGTTCTTAATGTAGCTGTTACAACTCCGACACACAGTTACGAATTAGTTCA
ATTACCATTTGGTAATCCAGTTTGGAATATATGGATGAATAATATTCATTATTCTACGTCAATATTGAATGA
TTATTTTAATGAAAAAATTAGATTGTGCACAGTTTATCCTCGACTTCTTAATCCTACTATTAATGATGCCA
TTACTATTACTTGGAATGGTATCATTCAATTTATCAGATCAGACGAATGGATCTTGATGTCTGGTGATCA
CATACATCATACGTATAGTATCTTTGTGAAATTGGTACAAAATAAGAAATTGGCTTTAAGAGTTTATATTG
GTGAACATGAAATGGAAATTATGCCTCTTGAAACAAAAGTATCTGTGAAAATAGATAATAAAATTATTG
ATGATTATCAAAAAGGTATTACAGTATCCGAGAACGAATCACGTTCTTATTCTATGAGATTAACCACTGC
TTATAATTATGTAGTAATTGATTCACAAACGATACCAGTTCATATTTTCTATAAAGTAGACAGTGTGACG
GTTTCTCTGCATACTAATCTTCAGAGTCAAGTAACTGGAATCTGTGGTAACATGGATGTTATGCATAAAG
ATGAAATTCCAGACATACATAGTGTTTCATATCTCTAATAAAATGAAATGAAATCATCTAATCATATTTTT
CATCTTATGAAACATGTTTAGTGTCATTATTTAGAATCGTTTAACAACAGTTATTGATTTTAATATTATAAT
TATATTAACAATCAATAATTTCTTTTTAGGATAATATGGAATGATGATGTAGTTACAAAGAATAAAAAAAT
AAAATTTTA
>LOC108004007
CGACGAGCCGTGAATTCCTCTTCTAAAATCACTCGCACCACTGACATTAACCGCGCTACAATTCGATTC

CTTGAACCTCGATGGCTTAAAAAGAATCTTTAACGGCCGCTTTCAACCAGCTCGTTCGACGTCAAAAT
TCGATGGTTGATCGATTTTTTTTACTGGCCATCATCCAACAAGTTCGGTGGCTGTCGAGGACGACGCTC
GCTCCACGAGCGGTCCACTATATTTAAATATAGTCGAAGATCGTCGTAGAGAGGATGGACGAGCGGCT
AGTCTTATGTGCGAGCCGCTGATAGCAGCCCGCTCCTCTCGGTGGAGCAGCAGCAGCAGCAGGGGCG
GTAGAGTGACAAGCAAAGTGATCGAGAGGTGAGCTCGAGCAGAACGAAGAGGAGTGGTGTCGATCC
CTGAGTGGGCTGTGAACAGGCCAGTCTCGTTTCAAGTGTACAACCCGGGCAAAAATCTTCAGCCGAA
GGGGGCTGTACGTCGGCCCGTTGCCGCAACGGGCTTCCTCTCTGCGCCGTTCGCCAACTGTCCGAGA
GATATGACGTCCGCCTGTTTCTCTCCTCCGCCGATCCGACCACCGATCGACCTCAACGATCAATGCGAC
ACAGTCAAGGGCACCGACTTGATCAAAGTGTCGAGGTTTTACAAGGATAGCAAGGAGGGAAAGTTCG
TGAGCTTCTTGTACGAGGACACGAGGACTCTTTACGACGGTTTCCGCAAAGGCGCCAAGGAGTCTAA
CAACGGTCCTTGCCTTGGCTGGCGGGATGGACCAAACAAACCGTATCAATGGCTACATTACAACGAGA
CCCTGCTCAGGGCGAAGAATTTCGGTTCTGGCCTAGTGTCTCTGGGGCTGATGCCAGGATCGCACACA
CTCATCGGTCTCTACAGTCAGAACTGTCCAGAATGGATCCTCACGGAGCAGGCTTGTTACACGTACTC
TTTGGTAGTAGTACCTTTATACGATACACTGGGCCCTGACGCGTGTGCCTTCATCATTAATCAAGCTGAG
ATCAATCTGGTCGTGGTCGAGAACGATACCAAGTGCAATTTGCTTCTCGACAAAGCGCCAAGATGCCT
ACGAAAATTGGTGGTGATAAAAGAAACAAGACAGACGACGAACCAGAGGGCGAAGAACCGTGGCGT
CGAGTTGCTCAAGTTCGACGACGTTGAGCGGCTGGGCGCCCAAAAGAATCACCCGGAAGTCCCCCCT
AAATCTACCGACCTGTGCACCATATCCTACACATCTGGGACGACCGGTAACCCGAAAGGCGTGATGTT
GACGCACCAAAATGTGATAGCTGGCATAAGCGCGGTTCTCGTTCAGTTGGGCGAGCACAAGCCCTCTT
ACAAGGACACGATGATCAGCTTCTTGCCTCTGGCGCACATGTTGGAACGTTGCTGCGAGAATGGCATG
TACATGGTGGGCGGCTCCGTAGGATTTTACAGCGGCGATATCAAAAGGCTGTCCGAAGACATGAAGGC
CTTGAGGCCTACTGTCATGCCTGCTGTGCCCAGGCTGTTGAATCGAATGTACGACAAGATCCAATCTGA
ACTTCAAAACTCATGCTTGAAAAAGCTGGTGTTCAGCCTGGGAATGCGGGCGAAGGAGGCAGAAATC
AAGAAAGGGATAATCAGGAACAACAGCATATGGGATAAACTGGTGTTCAGCAAGATCAAGGAATCAA
CAGGAGGGAGGGTGAGGCTGATGGTCGTTGGTTCCGCGCCTTTGGCGGGAAACGTTCTCACGTTCAC
GAGATGTGCACTGGGGTGTTTGATCGTCGAAGGATACGGCCAGACCGAGTGCTGTGCACCGATCACTC
TTACCGTTCAGGGTGATCATGTACCGGAACACGTAGGACCACCAGTCCCCTGTTGTTGCATTAAATTGG
TAGACGTTCCGGAAATGGAATACTATGCGAAGAAGAACCAAGGTGAGGTATGCGTAAAAGGCACCAA
TGTGTTCGTGGGCTACTTTAAAGATCCGGAAAGAACAGCTCAAGCTATCGATGAATTCGGTTGGCATC
ATACCGGTGACGTTGGCATGTGGTTACCTAATGGAACACTTAAAATAATCGATCGAAGGAAACACACC
TTCAAGCTTTCGCAAGGAGAATACATAGTTCCAGAGAAAATTGAGAATATTTATTTACGCAGTCAATAC
GTTCATCAAGTGTTTGTCCACGGGGAATCCCTAAAGTCGTGCGTTGTAGGAATAGTAATACCAGATGTA
GATGTCGTAAAATGTTGGGCAGTGGAGAACGGTATACCAGGAACGTTGAGCGTACTATGTGCTAATCC
ACAAGTCAAACAATTGATCATGGATGATATGCTTTCATGGGGAAAAGAGGCCGGTCTCAAATCTTTCG
AACAGGTGAAAGATATTTATTTACATCCAGATCCGTTCTCTATACAAAATGGTCTGCTTACACCGTCATT
GAAAATGAAACGACCTCAGTTAAAAGATTACTTCAAGCCACAGATAGAAGATCTCTATCGACATTTGG
ACTGATCAGACTGAGCATCGATTTTGATTCTGTTTTCATTAGCATCAGATACAAAAACGCGAAGAAGAA
TCGAGGCGTGTTTGCGTCGCATTTACAAAGAAATCATCGAATATTTATTATTACTCCGCGATATTTTTATT
ATATCATTTTTTTTCATTGATTACATATATAGCGACGCAAGAAAGAATAAAAAAAAAAAAACATCGTTTTT
AGATTATTAAAATATTGATAATGTTATAATAAAATGAATTATTATTAGGCGTCTCGTTTTCTCTTGACTATA
ATCATTTTTCGATACTTGTCACGTATATTTACAGTCTTCTTCGTTATCCCATCCTTGTATTTTGATACAAAT
TATTATCGCTTTTACTTAATGGTACACTATAGATGTATATTTTTATTGATAACAATAATTAATTAAATTTTGA
ATTATAATTTAATACTTTAAGATATGTATATTATTCGTTTCCGTAAAATGCAATCGATTGCGATATGTAAAA
CAAAACTTGAGGTTCACGTAATAAATTGATTGCAAAAACGGAGAATTTGAAAAGTAATAAAGATAATC

TCAAATA
>LOC107998473
TCGCCCACTGTTATTCGCGTGGTATTACGCGGTTTATATTAGTTCCCTAGATCGATCGATACAATTTTTAT
CAAAATCTTCATTTCCCATTTGAGAAAGAAGAGAGAACTAGATTAAGATCAAGTTGTTCTCCCTTCAA
AATGAAATATAATATTTTGATTTTTTGTATCGTTGGCGCTGCTCTCTGTGAATGTTCAATCGAGCAACCAT
TGGTCGAAGCCCCGATTGGGAAAATTCGCGGTTCAATTATCGTCTCAAGGCATGGGAGGAAGATTTATT
CGTTCCGTGGAGTGAGATATGGGGAGCCACCTGTCGGAAAGCAACGTTTTCAACCTCCAATCCCAGCG
GCGGATTGGCAAAACGTGTTCGACGCCACCGAGGAAGGACCTAGTTGCCCTCATCCCGATGGCGTGTT
GCAAGCGGAGGATTGTCTACGTTTGAACGTGTACACCACTAAACTACCGTGCGAGGAGAAAAATGTA
AAGAGACCAGTTATGATATTCATACATCCCGGCGGTTTTACCAGTTTCTCCGGGCAAAGTTCAATTTTC
GGCCCTCAATACCTACTCGACAAAGATATCGTATTGGTCACGATTAACTATCGTCTCGGGGCTCTAGGTT
TCTTGAATACAGGCGACAGTGAGGCACCCGGCAACATGGGCTTGAAGGATCAGGTAGAGGCGTTTAG
ATGGGTTCGAAGGAACATAGCCGCGTTCGGCGGCGATCCGAGTTCCGTCACGTTGTGCGGTTACAGCG
CGGGCAGTTTCAGCATAATGTTGCACATGGTCTCCCCGATGTCTAAAGATCTGTTCCACAGAGCTATAT
CGATGAGCGGCTCGGCCATCAAGCCGGAAGTCTACACAGGTGTCGCGGAACACGGGCAGAAGGAAC
TGGTTCAAAGGCAGGCACGGCTGTTGAATTGTCCCACCGATTCGACCGCTTCCATGTTGAACTGTTTG
ATCGAGAAACCCGTGGAAAACTTCACGAACACGTTGGCCAACTTGACGGATTGGTATGGAAATCCTAT
TCTTCTTTGGACACCAGCCGTGGAGCCCCAAGTCCCTGGTGTCGAACGATTCTTGTCCGAACAACCGT
ACGACTCGATCACGCAAGGGAAATTCCACCAAGTTCCTTACATACTCGGGGTCACGGAACACGAGTTT
GCCGGCGTTGCCGCGTTGTACGAGAGGAACGACAAGGTGGACAACGGGAGTTTGTATCGAGAGGTGA
ACAACAATTGGAACATGGTCGCCCCGATCTTTTGCATGTACGAACGCGATACTTCGCGATCGAATTACA
TAAGCAGGCAATTGAGACAGTTTTATTTCAAAAACGAGGCGATCAGCGAAACGACTCTCCTCCAACTT
GGCAAAGTATACGGCGATTGTATAACCATATTCCCCATGTATCGAGCGGTTAAATTGTTCGCTTCGAACT
CGAGAGAGCCGGTATACTTTTACAAATTTACGTACGAGGGACGATTCGGTTTCTACAGATGGAGCAAC
GATACGGCATACAATCCGTCGCATCACGACGATTTGCAGTATCTGTTTCACGCGAAACAATTCCCGTTT
CTCCCTTATTTGGAGGATGACGCACCGGAAGCGCCCATGGTTGAGCTTTACACGAGCATGTGGTCCAA
TTTCATAATAAACGGGGAACCGATCCCGAGGAACGATGACAGATTCAGAAACGTTTCGTGGGAGACGT
TCGACCCCTCGAAAACGAATTATCTGGAGATCGATCTTCGTTTGGGAATGAAAACTGAATTCTTCCCCG
AAAGGATGCGCTTATGGGAAAAATTATTCCCGTTGCCCTCTCAAGCTTCGAGAGGAGTGAAGCATTGA
ACATATATCTCTATTTTTCCACTGCGAAATTTCGATAAAATTATATACAACCTTTGATTGGAGGAATAAAA
ATAATTCTTCGAATTAAA
>LOC107998988
ATAGGTATTATTAAATTTAAAAAAAATTTCCTTTTTTACAATCAACGAGTGTGTACGTAACGTTTTATTTT
CGTTATATTATTTGGAAATATTCAACGCGCGCGATCATACGTAACGTATGTATCTAAAATAGATTCTTTCC
TTTTTTTAACAATGCTGTTAAAACTACCACTCTCGAGCATTCGAGAAATCGTGATCACACGAGGACAC
GACGAGGTATCTGAACCGCAGATATCGTGAGAATTGGCTTCGTTCGAACGGAGGCTCTCGATTAATTTA
ACGAATCCATCGGATAAACGCAGAGAGAGAGAGAGTGGATCGAAAATTCGGTTGAAAGCTGGGCTATCG
GTTATTGATTTTTCAGCCGGTTGTTGCAACAATGACAGTCGGTCAGCATTGTTGAGCGACAGGCATCGG
TAGGCAGCTTCAGGGAAAGGGGGGAGTTCGTTGAACGTTAGCTTCGGAGAATACGGTACACGGAGTA
TTGTGTAATATGAGTAATTGTTGCAGCATGTGATTGGTCACTGGTCGAGTCGATTAGAAAGAGAGAGA
GGGGGAGAGAGAGAAAAAGAAGTGTTTAAAAGTTTGAATAGAGAAAGAAGTGACAATGACAAGATT
TGAGTCATAAGTAAGAAAAAAAGACTTATTTAAAAACGAATATTCGACAAACTTTTATCGTTTGTAAAG
AATGAAACATCGATGACCGGAAGAACGTATTAAGTTGGAATAAAATTTTAAGACAAAAGAGAAAAGA
AACGATATACCGCTGTAATTTTTCTTACGCAAGGTGTGAAGGTATAAAATTATAAACAGAGCGATTCGA

CAGAGCGAATATTGGATGTCGGCCGTATGGCAGGGATGAATGGGCAACATCCGGTTTTCTGATTCACCA
AGATATTCTTGAGAAATGCGTAAGACGCACGCCTAGACGTTTGCTCGATCCAATATTGTCTTAACTGAT
GGATAATTGATAAAATCGAGCCGCTCTCGCCTTTTAACGTCGAGAAAAATAACCGCTGTTTTGCATCGT
TCAATCTCGTCACTACGAGATAAACGAAGATTGTTTCATCATTGCTTACAAATCGATCGATCGATGTATT
TGTATTTGTAAAAAAAAAAAAAAAAAAAGAGAGAATCGAGAAACGATCATTTACAAATTTTTTATCGTT
CACGGATGGAAAAGTATAGTACCGATGCAAACATGAGACAGTGTTTCGACGGAGTTTTAGTACACGGA
GGAACAAGCACTCCTTTAACTAATCACTATGTGAATGATGCAAATTATTATGAAATTTCAAGCAATGAC
AATTCAAGTCCCTTGGAGAAATTTTACTTAATTACTGGACAAAGGTGGCACACAGATACTGTGCTACCT
ATTCCTTTATTGCCATCTACAGAGGGAATGTCTGCTCGATCTCATCACAGATATCAACTTTTTATACATAG
GATCCCACAGTCCATAACGGGACTGGATGATGCTGGAATGGATTTGCAAGCAGCTCAACTCAGTACCC
GCACAGGACAGGAGGTACGTTCCACTGCAACCTTATCATGGCTAGATTGGCAATTGCCCCTGTTCGTA
GCAGTTTGCGCCATCGGTGGCGTCATTCTCTTTACATTTCTGATTTTGTTATGGCTGCGGAAACAAATGT
CGCGTGAAAAAGATACCGAAGATGGTGATAGGAGAGGTTTGGTAGAGGAAGGTGCTGTGGGTCATCC
GGAAAAATCTATAAAATCTTCAAAAGGATCGATTGAATGGGTTCATCAACCCGCAACGAAATCATCAG
TTCAATCTGTTCCTACTCGATCTCTTACGCAAGCCATTGGTTTACCAGAAGTAATGTCAGTAGCAACTC
CAGAACGCAGACCGGAACCGATACGCATAAGAACTAAAGGATTGTTAGAACGACGTGGATCAAGCGC
AAGTTTGACTATAGAATTGGCACCACCTCCAGAAAGTCCACCTCATATTGTTACACCTACTCGTGAATG
TACTGCAGAAGAATTTCTATTAAGCGCTGGAAATGTTTTATCAAGAGCACAACTTAGAAAGGCAATCA
GTGACACGGCATCCTTACATAAAGAATTTTGGGAAGTTCCTCTCAATTTACCCGAGAAATTAGATATATG
TGGATCTGGGGTGAAGAATAGATATTGTTCCGTATTACCAAATCCACAGTCAAGAGTGATTTTACCGGG
ACCTTCGGATGATCCTCTTTCGAGTTATATTAATGCCAATTATATTCGAGGATATGATGGAGAAGATGCA
CGCTATATTGCAACACAGGGACCATTAGCTCACACTGTGGATGATTTTTGGAAAATGATTTGGGCAGAA
AAAGTACCCGCGATAGTTATGATGACGAAATTATATGAAGCCGCCAAAACAAAATGCGAAGCATATTTT
CCATTGGATAAAAATAATCGCATACAGGCTGGACTGTTTACTATTATTGTTACTTCTATTGACACAAGAG
AAGGATATACGATCAGAGATTTAGAACTTCGATATGAAGGCGAAAGGAAGCATGTGCAGCATTACTGG
TATGATTCATGGCCAGACCATGCTGTACCTCAAACAGCAGATACCCTTGTTAGTTTAGCTGCAGAAGTA
AATTCTTTACCAGGTCCGGTCGTTGTTCATTGTAGTGCTGGAATTGGACGAACTGGTTGTTTCATAGCT
TTAGCTACGGGAATGACGCAGTTATTACGAGATGGGAATGTAGATGTATTGGGTATTTTATGCCAAATGA
GATATGACAGAGGTGGTATGGTTCAGACAGCAGAACAATACGAATTCGTTCATCGTGCACTTTGTCTGT
ATGAACAGACACTTGAAGGTGGTAAATCATCAAGTTCAGGTGATTGAAATTATAAACAAAGTGATTATG
GGAATTGATAAAAGTTCAGTAGAAGAAAAAGAATTGGTCTTAGAATTATTATGAAAAGATTCATGAAAT
GAATATGGTCTGCAACATATTACTAACGTCCGGGTGTGAATATTTGTATTATGGAGGAAATCCTATGAAA
AGAATCTCGATTTCAATTCTAGACCAGCGGATGTCTTACTCGATCTTCAGCTATTCAAGATGAGTTTTAT
CTCACTAAATAAGCTGCCATTTCTATACTGCCATGACAAATCGTTGACAATTAATATCAGTCACACAAAG
CTCAAGATAAAGGTAAATGGTTATTGACTCATGTTCTAAAGAGTTCTACATTATTCTTAAACGGAATTAA
AACTGTTTGACAAATGGAAAAAAAAAAAAAAAAGAAAAAGAAAAAAAAAGAAAAAAAATATATTTCAA
TTTGACTCGCGAGATATCAGGGTTTGTCAAAGTATTGATTCAGATTTTTATGCATTCTCTGTGTGTTATA
AATTAGATTTGTTAGTTATAAATATTTGCATCTATAATATTCTCAGAGTCCTATTCTTTCTACATTATGAGC
AGTTCTTACTTAGCTTTCCAGTACAGTAAACTATTACAAAACTAATATTGCTGTCTATTATACAATTACAA
AAATAATAACATTAATAGTTATAATTATTTCAAAATGAAGTCTTAAATATACAGAAGGGCAAATAATTATA
AAATTGGGACCCAGTTTAATTAAAAAATTTTTATCCTATTTGTAGTTCCTAGTTAATTTTACAGTAAAATG
TACACACAATGATTTTTTCCATGATTTTTAATTAATTTATCAATAAAGACTGAATGCAGGTAGTACAATAT
GTTTATTATATATTATAAGTTATAAATTTAACTAGAAATATTATGCTTCTCTGATACAAAGCAAAATTTATG
ATGTTATAAGATTGTAAAGAATATTATAGTCTTACTTGAAAAAAAGAAGAAAAAAGATAAATTGTAGAA

TGAGTTTGTTAAGCTTTAAAGCAAACTATTCGTTATATAGCAAAAGTAAAGTTGTTATAGCATTCTTTCC
AAGTATTATATATATACAAAACTATAATTATTTATTTTCTTTACTATATTTTGAATATGAATTATTAATTAATA
AAATTCCAATATAATGCTTTGAATTAATGATGAAAAAAATTTTTGATTCTTTTTTTTTAATAATGATCAA
TTTATTTGATTGAATATTAGTCTATAGTTGACAATAAGTGTGATTTATGAGCTTTTAGGATTTGATATGAAT
TTATTAACAAAAATGGCAATAGTTTGAACAGCAATGTTCAATTCTCTGCATAGAACTATTATAATTCAAT
TTTGACATAATAATACCAAAGTTATTAATATTCATTAATATTATATATTATAATGAGATATATTGCAGACTTT
ATAAGATTTATGATTAATAATTGTTGGAATTATAAAAGTTTGATTTATTTTTTTTATAATTAATTCTCACTG
AATTCCAATTTGAGTTATTATTATACATTAGGTTATGTATTTCGATTTCATTGTTTTGAATAATTTTTATAAT
TTGTGAATATATTGTAATTGAAAATTTAGCTTCATGTTATTTCAAAAATATAATCAATTTATTCATCGAAC
GTATTATAACATCATCATTCAAATTATTAATATAATGTTTTAGAATTCTTCAATCTTGTAAGTGAACAGTG
TGAACGTGTATTTTTGAGCGAAAGATTGTGATATGTATACATATATAATATATAATATATATATTTGTACATA
TGTTGTACAGAGAGTAAAATATATTTAAGAGATTATGTATTTGTGTAAGTGCAAATTGAGAGTATATTATT
TATGAGTATATAAAAGCCTTAAGCAACTTTAATGTAAACATATTAATAACGTAACAATAACAATAATAATT
ATTTAGAAACCCTTTGGTGTTCAATTGTACTTATGTGACGTAATAAATGTTTATAGTACCCATGCAA
