# Supplementary material for: Full-Length Transcriptome Analysis of Alternative Splicing and Polyadenylation in the Molecular Regulation of Labor Division in Apis cerana cerana
Source: Int J Mol Sci. 2025 Aug 14;26(16):7859. doi: 10.3390/ijms26167859 (PMC12387084; doi:10.3390/ijms26167859)
Supplement: Supplementary file 1 [file ijms-26-07859-s001.zip › Primers for PCR & 3í»RACE..pdf]

Table S1. Primers for PCR & 3'RACE.

Gene name	Primer sequence (5′- 3′)
XM_017060180.2	F: GTTTCCTGTGTGTACGTTAG<br>R: AGAGATTTCTTTGATTTTGA
LOC107998857_novel04	F: TTTACAGATTTTTCGCCATATA<br>R: GACACCAGCATTCACATCGG
LOC107998857	F: GTATCCGAGAACGAATCACG<br>R: AGTAACTGGAATCTGTGGTA
LOC108004007	F: GGTCTGCTTACACCGTCATT<br>R: ATCGCTTTTACTTAATGGTA
LOC107998473	F: CGGTTTCTACAGATGGAGCA<br>R: AACGATGACAGATTCAGAAA
LOC107998988	F: GTATTTCGATTTCATTGTTT<br>R: AAGAGATTATGTATTTGTGT
